# Discovery and *in vitro* characterization of a human anti-CD36 scFv

**DOI:** 10.3389/fimmu.2025.1531171

**Published:** 2025-02-04

**Authors:** Cecilia Mata-Cruz, Sandra L. Guerrero-Rodríguez, Keyla Gómez-Castellano, Gregorio Carballo-Uicab, Juan Carlos Almagro, S. Mayra Pérez-Tapia, Marco A. Velasco-Velázquez

**Affiliations:** ^1^ School of Medicine, Universidad Nacional Autónoma de México, Mexico City, Mexico; ^2^ Graduate Program in Biochemical Sciences, Universidad Nacional Autónoma de México, Mexico City, Mexico; ^3^ Research and Development in Biotherapeutics Unit (UDIBI), National School of Biological Sciences, National Polytechnic Institute, Mexico City, Mexico; ^4^ GlobalBio, Inc., Cambridge, MA, United States; ^5^ National Laboratory for Specialized Services of Investigation, Development and Innovation (I+D+i) for Pharma Chemicals and Biotechnological Products, LANSEIDI-FarBiotec-CONAHCYT, Mexico City, Mexico; ^6^ Immunology Department, National School of Biological Sciences, National Polytechnic Institute, Mexico City, Mexico

**Keywords:** CD36, scFv antibodies, cancer, atherosclerotic cardiovascular disease, phage display, human antibodies, lipid signaling

## Abstract

**Introduction:**

CD36 is a membrane receptor that participates in the cellular uptake of fatty acids and lipid metabolism. CD36 overexpression favors progression of different pathologies, such as atherosclerosis and cancer. Thus, CD36 targeting has medicinal relevance. Herein, we aimed to identify human anti-CD36 single-chain variable fragment (scFv) with therapeutic potential.

**Methods:**

The semisynthetic ALTHEA Gold Plus Libraries™ were panned using recombinant human CD36. Clone selection was performed by ELISA. Analysis of scFv binding and blocking function was evaluated by flow cytometry in macrophage-like THP-1 cells and hepatocellular carcinoma HepG2 cells. The phenotypic changes induced by CD36 ligands were assessed *in vitro* by: i) oil red staining, ii) tumorsphere assays, and iii) RT-qPCR.

**Results:**

We identified an anti-CD36 scFv, called D11, that competes with a commercial anti-CD36 antibody with proven efficacy in disease models. D11 binds to CD36 expressed in the membrane of the cellular models employed and reduces the uptake of CD36 ligands. In macrophage-like THP-1 cells, D11 impaired the acquisition of foam cell phenotype induced by oxLDL, decreasing lipid droplet content and the expression of lipid metabolism genes. Treatment of HepG2 cells with D11 reduced lipid accumulation and the enhanced clonogenicity stimulated by palmitate.

**Conclusion:**

We discovered a new fully human scFv that is an effective blocker of CD36. Since D11 reduces the acquisition of pathogenic features induced by CD36 ligands, it could support the generation of therapeutic proteins targeting CD36.

## 1 Introduction

CD36 is a membrane protein that is naturally expressed in different cell types including adipocytes, monocytes, macrophages, platelets, endothelial cells, cardiomyocytes, dendritic cells, epithelial cells, erythrocytes and muscle cells (1, 2). CD36 interacts with different ligands, including oxidized Low-Density Lipoproteins (oxLDL) (3) and fatty acids (FA) (4–6). The interactions of CD36 with these ligands trigger changes in lipid metabolism and signaling that drive disease progression.

For example, in atherosclerosis, a chronic progressive inflammation of the arteries walls, macrophages show increased uptake of oxLDL from blood using CD36 and other scavenger receptors, including LOX-1 and SR-A1 (7). Consequently, cholesterol esters are accumulated intracellularly in lipid droplets and macrophages are differentiated into foam cells, which play a key role in the formation of the early atherosclerotic plaque (8). Atherosclerosis favors the development of cardiovascular diseases, such as ischemic heart disease, heart failure, cerebral vascular disease and peripheral arterial insufficiency, that constitute the main cause of morbidity and mortality worldwide (9).

Similarly, CD36 is overexpressed in cancer cells, where it promotes changes in functions associated with tumor development and progression (reviewed by (10)). CD36-mediated signaling stimulates cell proliferation, migration, invasion and epithelial-mesenchymal transition (EMT), radioresistance, chemoresistance, clonogenicity, and stemness (11–19). These functions of CD36 have been reported for cancer cells from different tumor types, including hepatocellular carcinoma (HCC) (13, 20, 21), breast cancer (16, 22), bladder cancer (18, 23), stomach cancer (24, 25), ovarian cancer (26, 27), head and neck squamous cell carcinoma (14, 28), lung cancer (29, 30), and others (11, 17, 19).

Given the importance of CD36 in pathogenesis, it has been proposed as a therapeutic target in multiple diseases including atherosclerosis and cancer (10, 31, 32). As a proof of concept, anti-CD36 commercial antibodies of murine origin have been employed as CD36-blocking agents. For example, in a seminal work, Pascual et al. demonstrated that the anti-CD36 JC63.1 and FA6-152 antibodies inhibit lymph node and lung metastasis generated by the orthotopic implantation of oral squamous cell carcinoma cells (28). Therefore, development of human anti-CD36 antibodies that effectively block the uptake of lipid ligands would be promising in the treatment of the different pathologies where the importance of CD36 as therapeutic target has been demonstrated.

Antibody fragment formats have advantages in particular situations. For instance, single-chain variable fragments (scFv) have increased tissue penetration and access to cryptic epitopes due to their small size (approximately 27 kDa), which may be useful in cancer immunotherapy (33). In addition, the lack of a Fc region removes antibody effector functions such as antibody-dependent cellular cytotoxicity (ADCC), antibody-dependent cellular phagocytosis (ADCP), or complement-dependent cytotoxicity (CDC), reducing the risk of an immune cell activation that targets non-pathological cells and leads to on-target toxicity (34).

In this study, aiming to discover human anti-CD36 scFv with blocking activity, we panned ALTHEA Gold Plus Libraries™ (35) with recombinant CD36. These libraries consist of four synthetic variable regions of the light chain (VL) that were built with the human 3-20/4, 4-01/4, 3–11/4 and 1-39/4 scaffolds. The 4-01/4 scaffold has a long light complementarity-determining region 1 (LCDR1) loop and the other three (3-20/4, 3-11/4 and 1-39/4) have short LCDR1 loops. Within the scaffolds with short LCDR1 loops, 3-20/4 has the canonical structure class 6-1-1, whereas 3-11/4 and 1-39/4 have the canonical structure class 2-1-1, thus providing structural diversity to the set of VL scaffolds. As counterpart of the four VL libraries, a universal variable region of the heavy chain (VH) library built with the human 3-23/4 scaffold is used. The human genes that served as a template to design these VL scaffolds and the VH scaffold are the most prevalent in immune responses to diverse targets and have frequently been used as scaffolds to build antibody phage displayed libraries (35, 36). The ALTHEA Gold Plus Libraries™ have been successfully employed for obtaining human antibodies against multiple diverse targets, including SARS-CoV-2 (37) and Chikungunya virus (38).

After screening the outcome of the panning with CD36 with diverse assays, we obtained an anti-CD36 scFv, called D11, which effectively blocked the CD36-mediated uptake of oxLDL or palmitate in macrophage-like and hepatocarcinoma cells, respectively. Furthermore, analysis of the biological activity of scFv D11 showed that it reduces the acquisition of phenotypes associated with disease progression that are triggered by CD36 ligands. Thus, D11 seems to be a good lead candidate to develop anti-CD36 therapies for diverse pathologies.

## 2 Methods

### 2.1 Phage libraries panning

Nunc Maxisorb plates (Thermo Scientific, Cat. 278743) were coated with 50 µg/mL recombinant human CD36 (rhCD36) from Sino Biological (Cat. 10752-H08H) in PBS overnight at 4°C. The coated wells were washed three times with PBS and blocked with 3% skimmed milk in PBS (MPBS 3%) for 1 h at 37°C. The first round of panning was carried out by adding 150 µL per well of a mixture with 1 × 10^12^ virions/mL of each the four ALTHEA Gold Plus Libraries™ (35) separately and incubating for 2 h at 37°C. Afterwards consecutive washing steps with PBS and PBS-0.1% tween (PBST) were performed. Two additional rounds of selection were done with a reduced concentration (10 µg/mL) of rhCD36. CD36-specific phages were eluted using 1 mg/mL of TPCK-treated trypsin (Sigma-Aldrich, Cat. T1426) for 10 min. An additional elution step was performed with Glycine-HCl pH 2.2 at room temperature (RT). Phages from both elutions were mixed and amplified in *Escherichia coli* TG1. The amplified phages were rescued with Helper phage CM13K (ADL, Cat. PH050L). Colonies were picked out from 2xYT plates, incubated in 2 mL Nunc™ DeepWell plates (Thermo Scientific, Cat. 278743) containing 2xYT with glucose (1%) and carbenicillin (100 µg/mL), and grown overnight at 37°C. Expression of scFv was induced with isopropyl-b-D-thiogalactopyranoside (IPTG) 1 mM with overnight incubation at 30°C.

### 2.2 ELISAs

The scFv expression of IPTG-induced supernatants was quantified by ELISA, as described previously (39). Briefly, NUNC MaxiSorp plates (Thermo Scientific, Cat. 278743) were coated with Protein L (1µg/mL) in coating buffer (BioRad, Cat. BUF030C) overnight at 4°C. The plates were washed with MPBS-Tween 0.1% (MPBST). Dilutions of each IPTG-induced supernatant were added to the Protein L-coated wells and incubated for 1 h at RT. The specificity of the scFv in IPTG-induced supernatants was assessed by side-by-side ELISAs using plates coated with rhCD36 (5 µg/mL) or bovine serum albumin (BSA; [100 µg/mL]).

Positive and unique clones as assessed by Sanger sequencing were purified (see below) and assayed by ELISA against hrCD36 (5 µg/mL) to corroborate binding to the target. Further, competition with the commercial anti-CD36 antibody JC63.1 (Abcam, Cat. ab23680) was assessed by coating NUNC MaxiSorp plates with 5 µg/mL of JC63.1 in a coating buffer. Dilutions of the positive or an unrelated (P5E1A6, an anti-SARS-CoV2 binder (37)) scFvs were incubated with 5 µg/mL of hrCD36 30 min at RT. The mixtures were added to JC63.1-coated wells and incubated for an additional hour at RT. Wells without scFv were used as additional negative controls.

Binding was revealed with an anti-Myc HRP-conjugated secondary antibody (Abcam, Cat. ab19312). After washing steps, TMB (BD OptEIA, BD Biosciences, Cat. 555214) was added and the reaction was stopped with 1 M phosphoric acid. The ELISA plates were read at 450 nm with a correction at 570 nm.

### 2.3 Production and quality control of recombinant proteins

The scFvs were expressed in *E. coli* TG1 as described (38). Briefly, 150 mL of 2xYT with glucose (1%) and carbenicillin (100 µg/mL) were inoculated with an overnight culture. The culture was grown, and expression was induced with IPTG 1 mM. The culture was then incubated at 30°C overnight. Cells were harvested by centrifugation and the supernatant was filtered using a 0.22 µm membrane and pH was adjusted to 7.2.

Purification of scFvs from IPTG-induced supernatants was performed by fast protein liquid chromatography (FPLC) using HiTrap Protein L (Cytiva, Cat. 17547815). The column was sanitized with 15 mM of NaOH and subsequently equilibrated with 5 column volume (CV) of binding buffer (20 mM Na_2_HPO_4_, 150 mM NaCl, pH7.2). Column flow was set to 3 mL/min. Finally, the column was washed until the signal returned to the baseline produced by the binding buffer. Elution was performed with acetic acid [0.1M, pH 2.8] and neutralization was done with Tris-HCl [1M, pH 9]. The neutralized buffer was exchanged by using Amicon Ultra-15 10kDa and PBS.

The concentration of the isolated scFv was quantitated by UV/VIS spectrophotometry on an Epoch System (Bio Tek Instruments), using the extinction coefficient 1 M^-1^cm^-1^. The structural integrity was assessed by denaturing SDS-PAGE using Any kD™ TGX Stain-Free™ Protein Gels (Bio-Rad, Cat. 4568123) and a Mini-PROTEAN^®^ system (Bio-Rad). scFv samples (2 μg/lane) were analyzed under non-reducing and reducing conditions. Data was acquired using the ChemiDoc Imaging Systems (Bio-Rad).

### 2.4 Cell culture

THP-1 (Cat. TIB-202) and HepG2 (Cat. HB-8065) cell lines were obtained from ATCC. THP-1 cells were cultured in RPMI-1640 supplemented with 10% Fetal Bovine Serum (FBS), 50 µM β-mercaptoethanol, penicillin (100 U/ml), and streptomycin (100 μg/ml). THP-1 cells were differentiated into macrophage-like cells by treating them with 200 ng/ml PMA for 5 days as reported (40). HepG2 cells were cultured in EMEM supplemented with 10% FBS, penicillin (100 U/ml), and streptomycin (100 μg/ml). All cell cultures were maintained at 37°C with 5% CO_2_ unless otherwise indicated.

### 2.5 Binding of scFv to membrane CD36

CD36 expression in PMA-treated THP-1 cells and HepG2 was validated by flow cytometry. 100,000 cells per sample were incubated for 45 min at 4°C with 1 µg/mL of the anti-CD36 antibody FA6-152 (Stem Cell, Cat. 60084). Detection was performed by adding anti-mouse Alexa Fluor 488 (Invitrogen, Cat. A21202) and the signal was compared with that generated by staining with the secondary antibody alone.

To quantify the binding of the scFvs to cell-expressed CD36, different concentrations of D11 or the unrelated scFv (P5E1A6) were incubated with the CD36 expressing cells during 45 min at 4°C. Binding to cells was detected with anti-His-tag PE antibody (R&D systems, Cat. IC050P) at 2 µg/mL. For all the assays, at least 10,000 events per sample were acquired using Attune NxT flow cytometer (Thermo Fisher). Experiments were done two independent times, each with duplicates.

### 2.6 OxLDL uptake assay

40,000 PMA-treated THP-1 cells were incubated with different concentrations of the positive and unique scFvs, P5E1A6, or anti-CD36 JC63.1 for 1 h at 37°C. Subsequently, oxLDL-DyLight 488 (Cayman, Cat. 601181) was added according to the manufacturer’s instructions. After incubation for 14-16 h, cells were washed with 1% BSA DPBS, detached with trypsin, and transferred to “V” bottom plates. Cells were stained with 7-AAD (Cayman, Cat. 601181) according to the manufacturer’s instructions. Internalization of oxLDL-DyLight 488 was analyzed by flow cytometry using an iQue3 instrument (Sartorius). Response was normalized using as 100% the MFI of untreated cells and as 0% the MFI of cells without oxLDL-DyLight 488. Three independent experiments were performed.

### 2.7 Palmitate uptake assay

200,000 HepG2 cells were exposed to scFv D11, scFv P5E1A6, or anti-CD36 FA6-152 for 1 h, and subsequently incubated with 50 ng/mL BODIPY-Palmitate (Cayman, Cat. 26749) for 15 min at RT. Cells were washed with 1% BSA DPBS, and stained with 7-AAD (Cayman, Cat. 601181) according to the manufacturer’s instructions. Internalization of BODIPY-Palmitate was quantified, acquiring 20,000 events per sample in FACS Aria III (BD Biosciences) or Attune NxT (Thermo Fisher) flow cytometers. The experiments were performed three independent times.

### 2.8 Oil red-O staining for lipid droplets

Macrophage-like THP-1 cells were treated with recombinant scFvs D11, scFv P5E1A6 (100 μg/ml), or anti-CD36 JC63.1 (50 μg/ml) for 1 h at 37°C. Subsequently, 50 μg/ml ox-LDL (Kalen Biomedical LLC, Cat. 770252-7) was added to the cultures for 48 h to generate foam cells. HepG2 cells were treated with recombinant scFvs D11, scFv P5E1A6 (100 μg/ml), anti-CD36 JC63.1, or anti-CD36 FA6-152 (50 μg/ml) for 1 h at 37°C before the addition of 50 mM sodium palmitate (Sigma-Aldrich, Cat. P97676). Cells were incubated for 16-18 h.

Lipid droplet quantification was performed in fixed cells (10% formalin for 1 h). Samples were washed with 60% isopropanol for 5 min and allowed to dry completely. Subsequently, a solution of Oil Red-O/0.5% isopropyl alcohol (Sigma-Aldrich, Cat. 01391) was added and incubated for 10 min. Samples were washed several times with distilled water before image acquisition in a microscopy (Eclipse Ti-U, Nikon). Stained area from at least seven fields with equivalent number of cells was quantified using imageJ (41) and averaged. Two independent experiments were performed.

### 2.9 Tumorsphere formation assay

The assay was performed as previously described (42). Briefly, 100 viable cells were plated on a 96-well ultra-low attachment plate (Corning Costar, Cat. 3473) with MammoCult medium and growth factors (StemCell Technologies, Cat. 05620) and cultured in presence of 50 μM palmitate prepared as previously reported (28). The cells were treated every 72 h with the recombinant scFvs or the commercial anti-CD36 JC63.1 for 7 d. The number of tumorspheres with diameter >60 μm was quantified by taking micrographs (Eclipse Ti-U microscopy, Nikon) and analyzing them in NIS Elements Basic Research software (Nikon). Sphere forming efficiency was calculated as reported (42) in three independent experiments with at least three replicates each.

### 2.10 Real-time PCR

Macrophage-like THP-1 or HepG2 were exposed for 16 h to oxLDL or palmitate, respectively. Total RNA was extracted using the RNeasy Plus Mini Kit (Qiagen, Cat. 74134). Real-time PCR were performed in triplicate using the QuantiTec SYBR Green RT-PCR kit (Qiagen, Cat. 204243) and the primers listed on **Supplementary Table S1**. Relative mRNA expression was calculated by 2^−ΔΔCt^ against β-actin expression (43). Two to three independent experiments were performed.

### 2.11 Statistical analysis

Statistical analyses were made using one-way analysis of variance (ANOVA) and Bonferroni´s *post hoc* analysis, using Graph Pad Prism 10 (Version 10.3.1). Statistical significance was set at p < 0.05.

## 3 Results

### 3.1 Discovery of scFv clones targeting CD36

**Figure 1:**
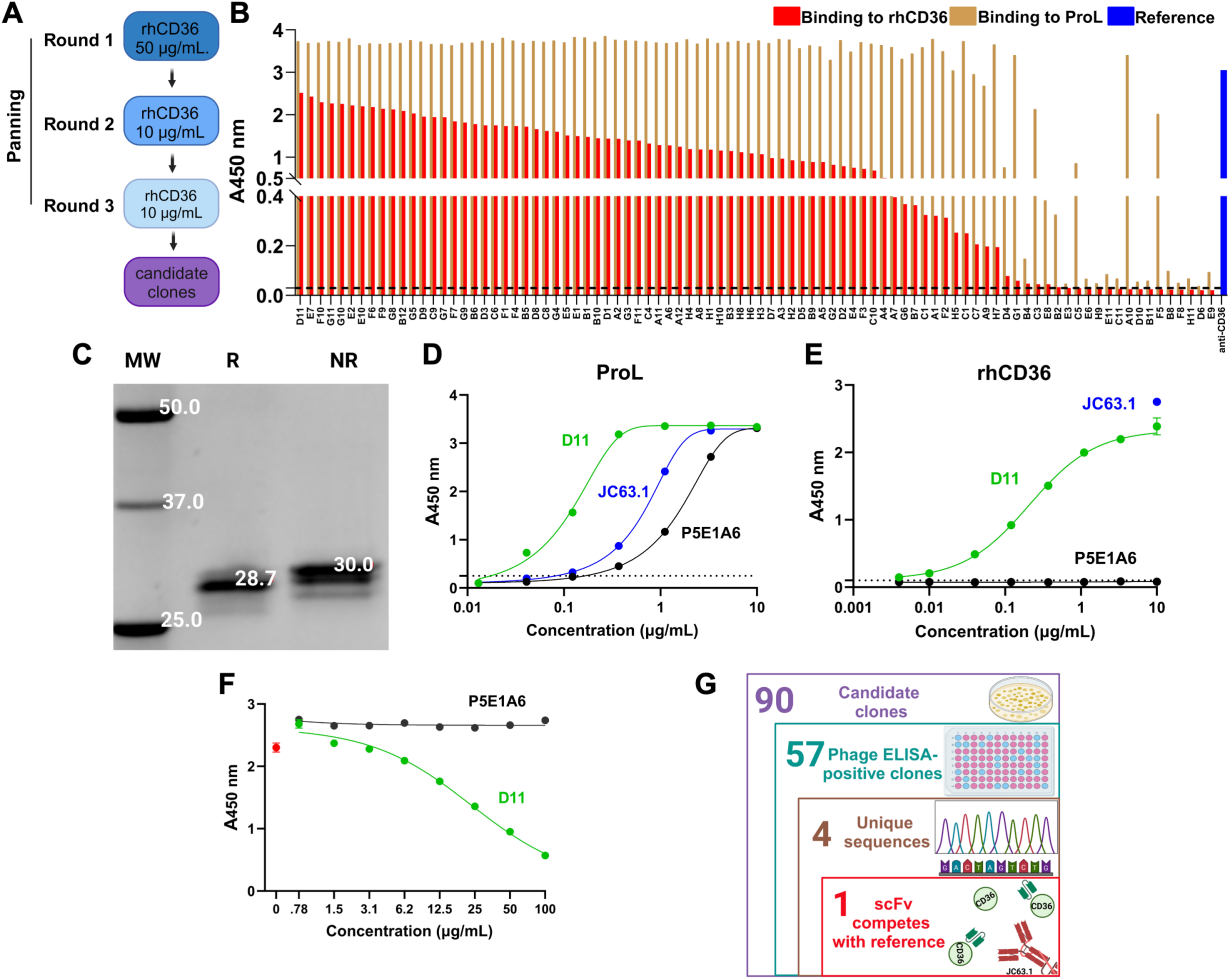
Identification of the scFv anti-CD36 D11 **(A)** Strategy of panning of the ALTHEA Gold Libraries™ for identification of anti-CD36 scFvs. **(B)** Phage ELISA for 90 randomly selected clones from the third round of panning. Graph shows the signals generated by binding to rhCD36 (red) or protein L (ProL; brown). As control, we detected rhCD36 with the commercial antibody JC63.1 (blue). **(C)** Determination of structural integrity of purified scFv D11 by denaturing SDS-PAGE. R, reducing condition; NR, non-reducing condition; MW, molecular weight. **(D, E)** ELISA assay evaluating the binding of purified D11 (green) to protein L **(D)** or rhCD36 **(E)**. JC63.1 (blue) was used as a positive control and specificity was assessed by using the unrelated scFv P5E1A6 (black). **(F)** Representative competition ELISA (from two performed) evaluating the binding to rhCD36 captured by the reference antibody JC63.1 in presence of D11 (green) or the specificity control P5E1A6 (black). The signal generated in the absence of D11 is shown in red. **(G)** Number of clones identified at each stage of the discovery process. **(G)** was created with Biorender.com.

We screened ALTHEA Gold Plus Libraries™ aiming to identify fully human anti-CD36 scFvs (**Figure 1A**). After three sequential rounds of solid panning using rhCD36 as a selector, we collected 90 random clones from the last round. Supernatants from IPTG-induced scFvs clones were subjected to three primary assays: i) rhCD36 binding; ii) protein L binding for assessing scFv expression; and iii) BSA binding to determine whether or not the positive clones were specific for CD36.

We identified 57 positive clones for hrCD36 and negative for BSA, with protein L binding (**Figure 1B**; **Supplementary Figure S1**). Sanger sequencing of those clones identified four unique scFvs. The clone with the highest ELISA signal for CD36 and that was found more than once, called D11, was expressed and Protein-L purified. Further characterization of D11 showed a single band of the expected molecular weight (**Figure 1C**), bound protein L (**Figure 1D**) and rhCD36 (**Figure 1E**). Binding to rhCD36 was consistent among the different batches of purified D11 (**Supplementary Figure S2**). Importantly, D11 blocked the binding of rhCD36 to the reference antibody JC63.1 to in a dose-dependent manner (**Figure 1F**) and the probable binding modes (**Supplementary Figure S3**), mapped by molecular docking, showed interactions with CD36 in close proximity to the reported oxLDL binding region (residues 157-171 (44)). Therefore, D11 seems to bind an epitope similar to that of the reference antibody JC63.1 (**Figure 1G**).

### 3.2 CD36 binding and blocking functions of D11 in cell-based assays

**Figure 2:**
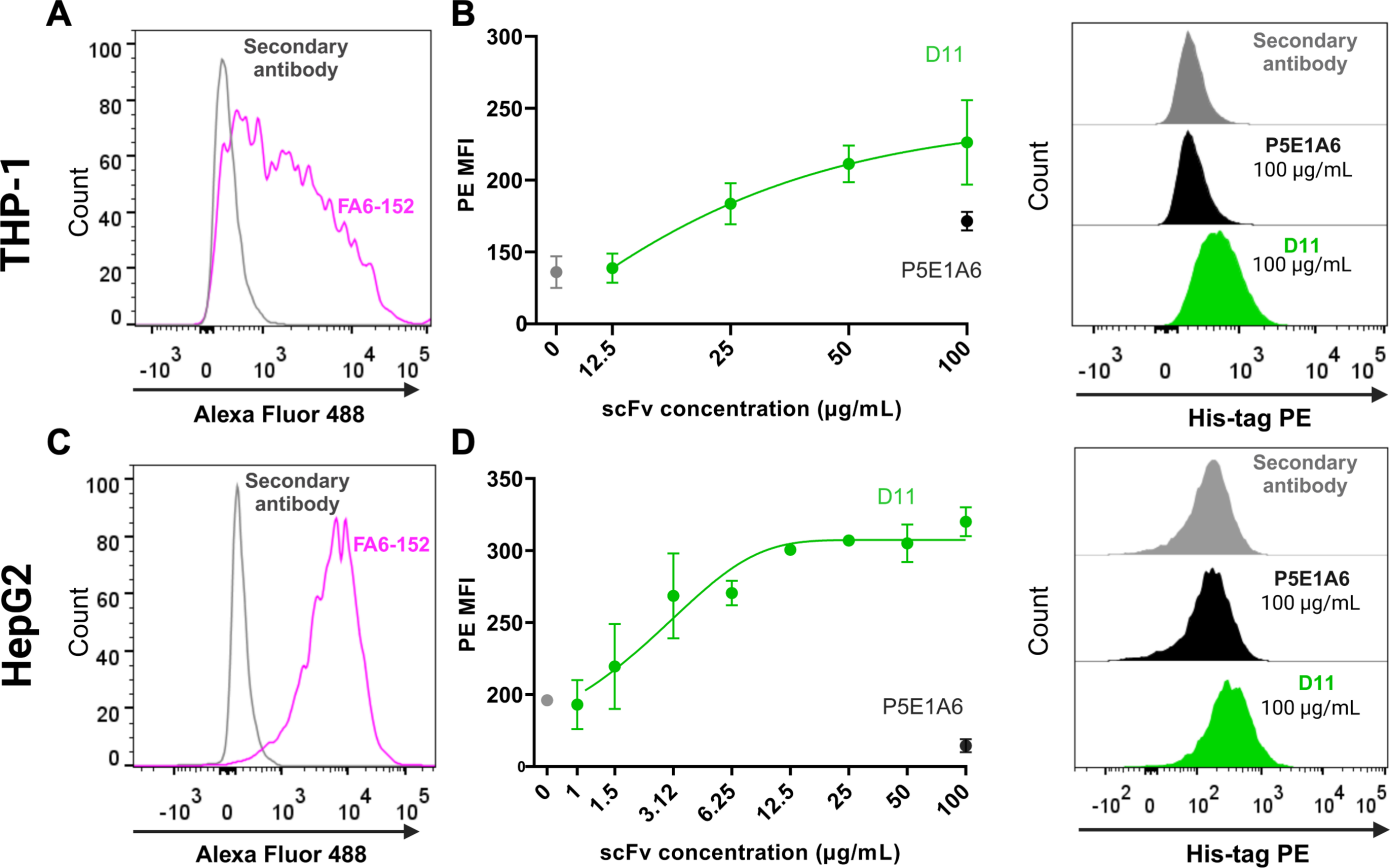
Binding of D11 to membrane CD36. **(A)** CD36 expression in macrophage-like THP-1 cells, as detected with the anti-CD36 FA6-152 (pink). The fluorescence generated by staining with the secondary antibody alone is shown in gray. **(B)** Concentration-response curve evaluating the binding of D11 to CD36^+^ macrophage-like THP-1 cells. Data from two independent experiments. Representative histograms are shown on the right. **(C)** CD36 expression in HepG2 cells. **(D)** Binding of D11 (green) to HepG2 cells and representative histograms (right). Data are representative of two independent experiments, each one including duplicates. **(B, D)** show the signals from the secondary antibody anti-His-tag PE (gray) and the unrelated scFv P5E1A6 (black).

Binding of D11 to mCD36 was assessed *in vitro* using two different cell-based assays using macrophage-like (PMA-treated) THP-1 cells or HepG2 hepatocarcinoma cells. Both cellular models displayed high mCD36 expression, as detected by flow cytometry using the commercial anti-CD36 clone FA6-152 (**Figures 2A, C**). D11 bound in a dose-dependent manner to mCD36 in macrophage-like THP-1 cells (**Figure 2B**) and HepG2 cells (**Figure 2D**). In contrast, the unrelated P5E1A6 did not show relevant binding to the cells (**Figures 2B, D**).

**Figure 3:**
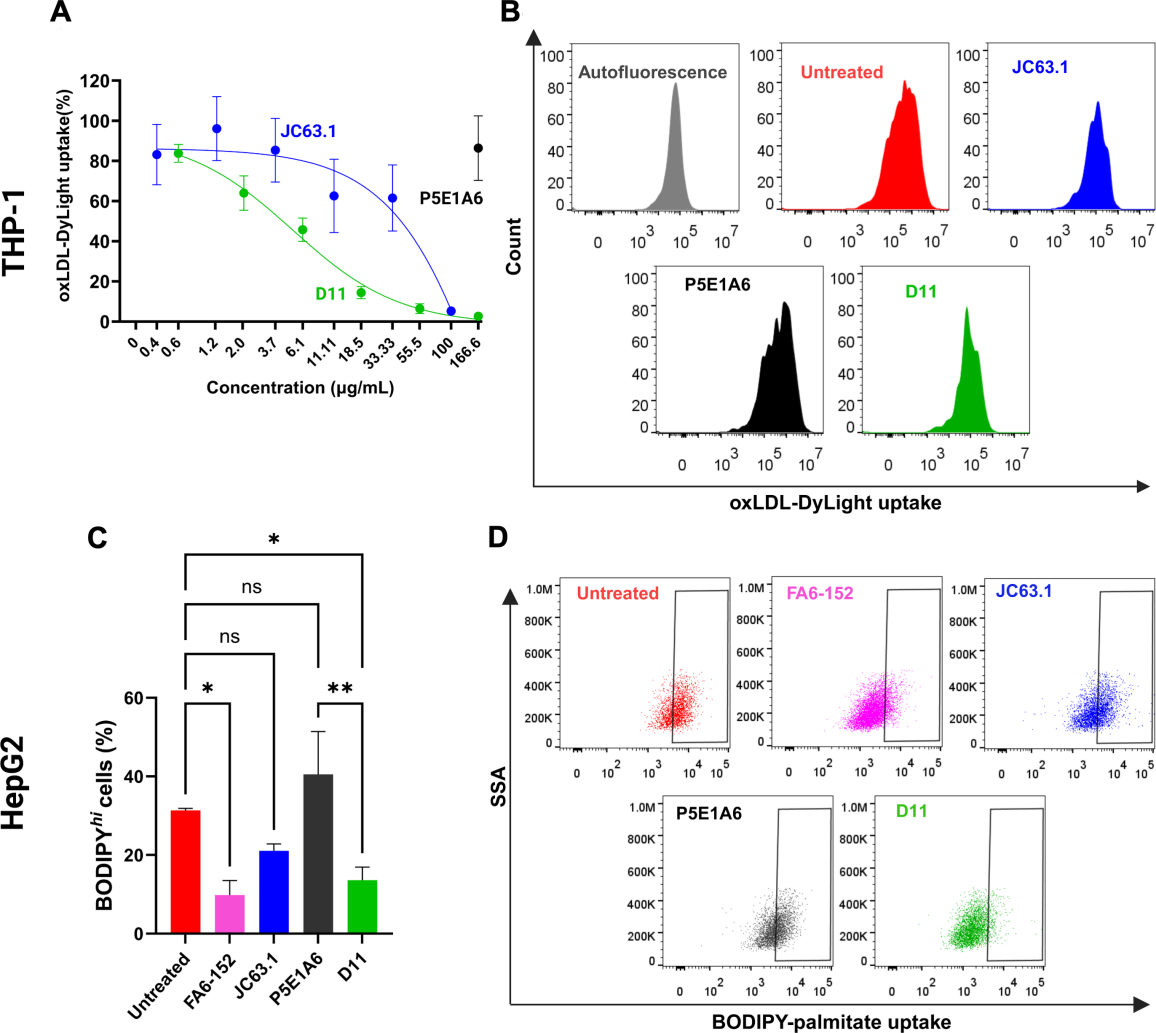
Effect of D11 in the uptake of CD36 ligands. **(A)** Analysis of oxLDL-DyLigth uptake in macrophage-like THP-1 cells. Cells were treated with D11 (green), JC63.1 (blue), or the unrelated P5E1A6 (black). Response was normalized against the MFI of untreated cells. Data from three independent experiments. **(B)** Representative histograms of the effect of the different treatments (100 µg/mL) on the uptake of oxLDL. **(C)** Effect of the listed antibodies (100 µg/mL) on the uptake of BODIPY-palmitate in HepG2 cells. Bars in the graph show the percentage of cells with BODIPY high fluorescence from three independent experiments (mean ± SEM). *p < 0.05; **p < 0.01, ns: not significant, Bonferroni’s multiple comparisons test. **(D)** Representative dot plots of the experiments reported in **(C)**.

The anti-CD36 JC63.1 is a reported blocker of CD36-mediated oxLDL transport (28). Since D11 was selected based on its ability to compete with JC63.1, we hypothesized that both antibodies could also share CD36-blocking activity. In macrophage-like THP-1 cells, the uptake of oxLDL-Dylight was inhibited by JC63.1, but was insensitive to P5E1A6 (**Figures 3A, B**), indicating that the employed system is specific for CD36-mediated activity. D11 blocked oxLDL-Dylight transport in a dose-dependent manner, achieving a maximum inhibitory response comparable to that generated by JC63.1 (**Figures 3A, B**).

Since the reported sites on CD36 for oxLDL and FA binding partially overlap (3, 4, 45), we also analyzed the effect of D11 on the CD36-mediated uptake of palmitate by HepG2 cells, as reported (20, 46, 47). D11 decreased the percentage of HepG2 cells with high uptake of BODIPY-palmitate (**Figures 3C, D**). The reduction generated by D11 was similar to that of the antibodies JC63.1 and FA6-152, a well characterized anti-CD36 with inhibitory effect on FA transport (28, 48). As expected, no effect of P5E1A6 was observed (**Figures 3C, D**). Altogether, these results demonstrate that the D11 binds to mCD36 with functional consequences.

### 3.3 The scFv D11 reduced the phenotypic changes induced by CD36 ligands

**Figure 4:**
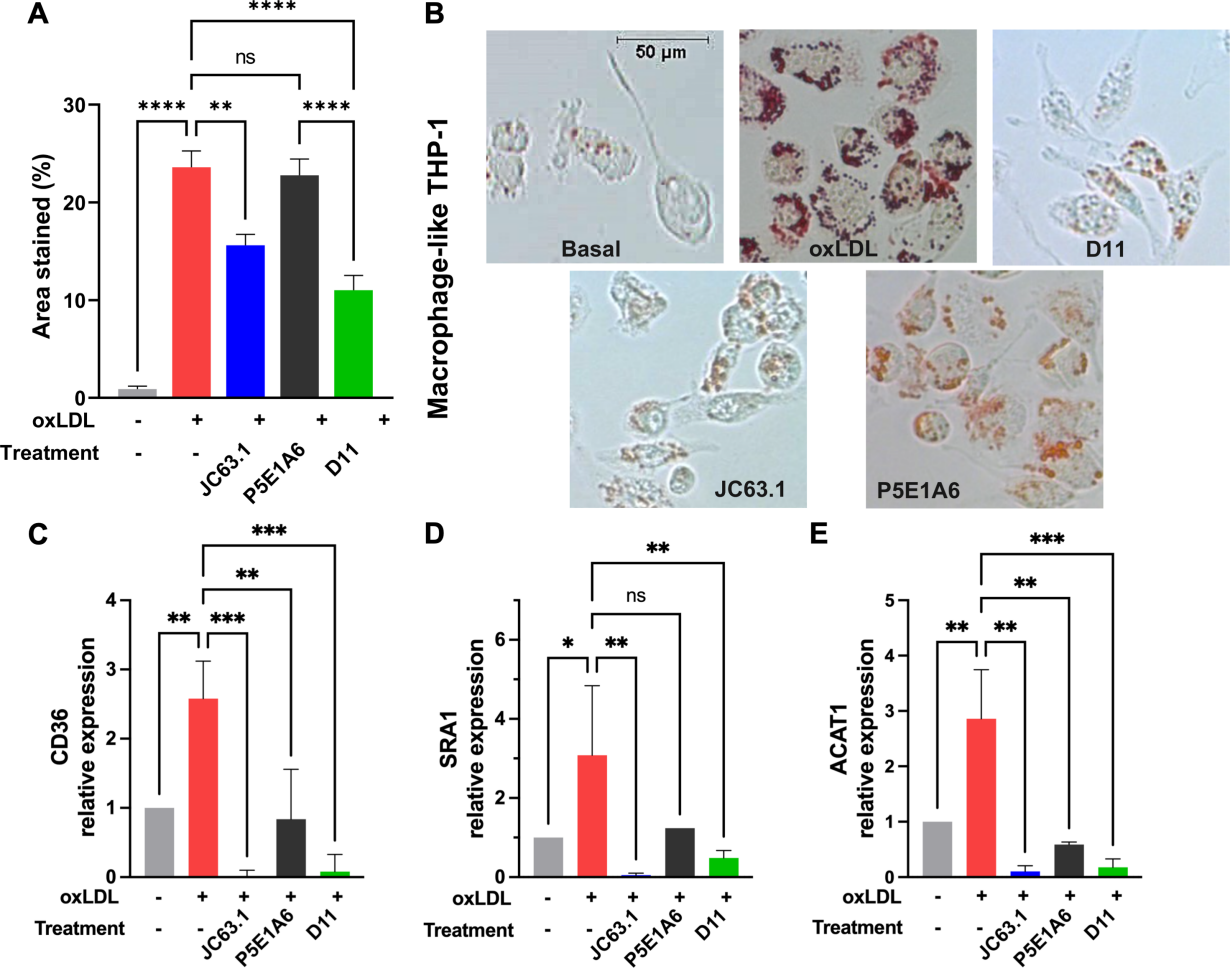
Effect of D11 on the oxLDL-induced foam cell phenotype. **(A)** Effect of treatments on LD accumulation in macrophage-like THP-1 cells exposed to oxLDL (50 μg/ml) for 16-18 h. Graph shows the quantification (mean ± SEM) of oil red staining from two independent experiments. **p < 0.01; ****p < 0.0001, Bonferroni´s multiple comparisons test. **(B)** Representative micrographs of the experiments reported in **(A)**. **(C-E)** Effect of treatments on the relative expression of CD36 **(C)**, SRA1 **(D)**, and ACAT **(E)**. Graphs show the geometric mean ± geometric SD from three independent experiments. *p < 0.05; **p < 0.01; ***p < 0.001, ns: not significant, Bonferroni´s multiple comparisons test.

To analyze the effect of D11 on a disease-relevant phenotype, we induced the differentiation of macrophage-like THP-1 to foam cells. The addition of oxLDL induced the accumulation of lipid droplets (**Figures 4A, B**), as reported (49–51). The coincubation with D11 or JC63.1 with oxLDL significantly decreased lipid droplet formation (**Figures 4A, B**). Foam cells show increased expression of genes participating in lipid metabolism, including CD36, ACAT, SRA1 and LOX-1 (52–54). In oxLDL-treated cells, we found increased mRNA levels of CD36, SRA1, and ACAT (**Figures 4C–E**), but not that of LOX-1 (**Supplementary Figure S4**). D11 abrogated such increases (**Figures 4C–E**), corroborating its blocking of CD36-mediated functions. Although P5E1A6 did not reduce the area stained by oil-red, it reduced the expression of CD36 and ACAT, suggesting that the expression of these genes is not exclusively controlled by CD36 in the cellular model employed.

**Figure 5:**
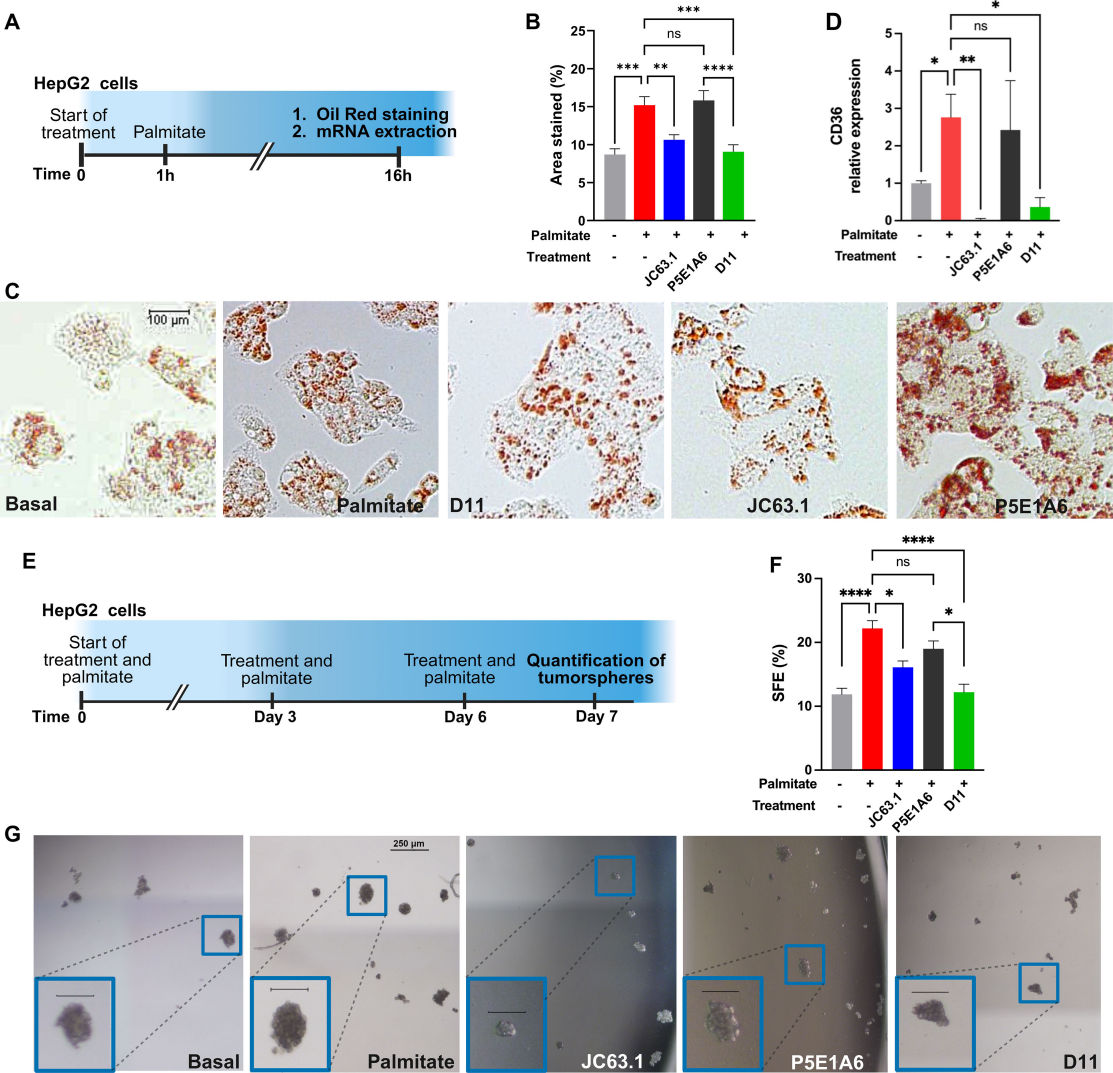
D11 impairs LD accumulation and clonogenicity on HepG2 cells. **(A)** Experimental protocol for the analysis of phenotypic changes induced by CD36 blockage. **(B)** Effect of treatments on LD accumulation. The bars in the graph (mean ± SEM) show the oil red-stained area from two independent experiments. **p < 0.01; ***p < 0.001; ****p < 0.0001, ns: not significant, Bonferroni´s multiple comparisons test. **(C)** Representative micrographs of the results presented in **(B)**. **(D)** Effect of treatments (100 µg/mL) on CD36 mRNA expression. Graph shows the geometric mean ± geometric SD from two independent experiments. *p < 0.05; **p < 0.01, ns: not significant, Bonferroni´s multiple comparisons test. **(E)** Experimental protocol for the evaluation of the effect of scFv D11 on the clonogenicity of HepG2 cells. **(F)** Effect of treatments on sphere formation efficiency (SFE). Data (mean ± SEM) from three independent experiments. *p < 0.05; ****p < 0.0001, ns: not significant, Bonferroni´s multiple comparisons test. **(G)** Representative micrographs of the results presented in **(F)**, with insets showing enlarged images of tumorspheres (scale=100 µm). **(A, E)** were created with Biorender.com.

The functionality of D11 as an inhibitor of palmitate-induced phenotype was evaluated in HepG2 hepatocarcinoma cells. Cells were exposed to anti-CD36 or control antibodies in presence of palmitate for 16 h (**Figure 5A**). In absence of additional treatment, palmitate increased the accumulation of lipid droplets (**Figures 5B, C**) and CD36 expression (**Figure 5D**). Those effects of palmitate were reduced by D11 and JC63.1, but not by the control P5E1A6 (**Figures 5B–D**). We also evaluated changes in other genes reported to be transcriptionally activated by CD36 signaling, but we found no effect of D11 treatment (**Supplementary Figure S5**).

Since CD36 promotes stemness in multiple cancer models (30, 55, 56), we analyzed the activity of D11 on HepG2 clonogenicity, using tumorsphere assays (**Figure 5E**). The augmented sphere forming efficiency (SFE) generated by palmitate, was reverted by JC63.1 and D11, but not by the unrelated negative control P5E1A6 (**Figures 5F, G**).

Our results, taken together, indicate that the newly discovered scFv D11 is an effective blocker of CD36 that reduces the acquisition of pathogenic features induced by lipid ligands.

## 4 Discussion and conclusion

In the previous sections we reported the selection and biological characterization of a human anti-CD36 antibody fragment, D11, from the ALTHEA Gold Libraries™. These libraries have been designed to increase the diversity and pharmaceutical developability of the antibody fragments selected from them and have been employed in the discovery of diverse antibodies for viral proteins and human soluble targets (38, 39, 57). Our phage panning campaign followed the technical considerations for selecting hits that could be further developed into antibody-based therapeutics (58). The selection screening of D11 included the ability to block CD36 binding to a well-known antibody, JC63.1. The latter antibody was used as a reference due to its inhibitory activity on oxLDL uptake in cellular models (18, 48) and its anti-metastatic effect *in vivo* (28). Selection by competition assays is a common strategy in antibody discovery (59, 60) since it is expected that antibodies competing for similar epitopes could have similar functional and biological activities (61, 62). As we hypothesized, D11 was an effective blocker of CD36-mediated uptake of oxLDL in CD36^+^ cells. Further, D11 was also an effective blocker of palmitate uptake, a finding supported by the partial overlap of the binding sites or both ligands (3, 4, 45). Moreover, it has been reported that those two ligands cooperate for CD36 activation (63). Finally, the potential therapeutic effects of D11 were demonstrated in two different models: oxLDL promotion of differentiation to foam cells and palmitate-mediated induction of aggressive features in hepatocarcinoma cells.

Foam cell differentiation from macrophages is a key step in the initiation and progression of atherosclerosis (64, 65). We established that D11 reduces the oxLDL-induced increase in lipid droplet content. We also observed decreases in the expression of target genes of the pathway activated by CD36, including CD36 −in a positive feedback loop−, ACAT, responsible for FA esterification, and SR-A1, another oxLDL transporter (53).

Similarly, D11 decreased lipid droplet biogenesis induced by palmitate in the hepatocarcinoma cells HepG2. The role of CD36 in cancer progression and aggressiveness has been extensively documented. In the case of HCC, analysis of The Cancer Genome Atlas (TCGA) Program showed that CD36 mRNA is highly expressed in HCC tissue and HCC cell lines (20). In hepatocarcinoma cells, CD36 expression favors the expression of EMT markers (46) and, as reported for other tumors, metastatic capability (21). On the other hand, CD36 knockdown in the HCC cell lines SMMC-7721 and HepG2 upregulates FA β-oxidation, activates the lipophagy pathway, and reduces hepatic lipid accumulation (66), supporting a key role of CD36 in lipid metabolism required for HCC progression.

Bort et. al. (13), demonstrated that alterations on lipid metabolism, including increased CD36 expression, improve lipid content accumulation and promote stemness in sorafenib-resistant HCC cells; thus, targeting lipid accumulation and biosynthesis reduces the cancer stem cell pool. Therefore, we decided to analyze the effect of D11 on the clonogenicity of HepG2. Our findings show that palmitate drastically increased the SFE in HepG2 cells, in agreement with previous reports (67). D11 abolished the tumorsphere-promoting effect of palmitate, indicating that metabolic reprogramming mediated by CD36, via increased uptake of exogenous FA, can be effectively blocked by D11.

Although further characterization of the D11 binding site and the phenotypes induced by it are still required, our results demonstrate that it can effectively modify lipid signaling pathways.

Since CD36 is broadly expressed and mediates multiple functions among vertebrates (68), an anti-CD36 therapeutic antibody should have blocking activity but minimal effector functions so that the relationship between efficacy and toxicity (therapeutic window) be maximized. Accordingly, we decide to characterize the biological effects of D11 without conversion to IgG, the therapeutic format of choice for antibodies. Our results validate the potential use of scFv D11. However, the *in vivo* short half-life of scFvs may be a drawback for D11 and thus, conversion to other therapeutic formats (69–71) or its coupling to a drug delivery system (72) should be considered as part of D11 therapeutic development program.

In conclusion, we discovered a human anti-CD36 scFv, D11, which blocks the uptake of CD36 ligands and reduces the acquisition of cellular phenotypes associated with disease progression. Therefore, D11 seems to be an excellent lead for generation of therapeutic proteins targeting CD36.

## Data Availability

The datasets will be available by request to the corresponding authors.
