# Supplementary material for: Discovery and in vitro characterization of a human anti-CD36 scFv
Source: Front Immunol. 2025 Feb 4;16:1531171. doi: 10.3389/fimmu.2025.1531171 (PMC11832482; doi:10.3389/fimmu.2025.1531171)
Supplement: Supplementary file 1 [file DataSheet1.pdf]

Discovery and in vitro characterization of a human anti-CD36 scFv

Cecilia Mata-Cruz[1,2], Sandra L. Guerrero-Rodriguez[1,2], Keyla Gómez-Castellano[3], Gregorio Carballo-Uicab[3], Juan Carlos Almagro[3,4], S. Mayra Pérez-Tapia[3,5,6] *, and Marco A. Velasco-Velázquez[1] *

[1]School of Medicine, Universidad Nacional Autónoma de México, Mexico City, Mexico

[2]Graduate Program in Biochemical Sciences, Universidad Nacional Autónoma de México, Mexico City, Mexico

[3]Research and Development in Biotherapeutics Unit (UDIBI), National School of Biological Sciences, National Polytechnic Institute, Mexico City, Mexico.

[4]GlobalBio, Inc., Cambridge, MA, USA

[5]National Laboratory for Specialized Services of Investigation, Development and Innovation (I+D+i) for Pharma Chemicals and Biotechnological products, LANSEIDI-FarBiotec-CONAHCYT, Mexico City, Mexico

[6]Research and Development in Biotherapeutics Unit (UDIBI), National School of Biological Sciences, National Polytechnic Institute, Mexico City, Mexico.

[7]Immunology Department, National School of Biological Sciences, National Polytechnic Institute, Mexico City, Mexico

Supplementary Information

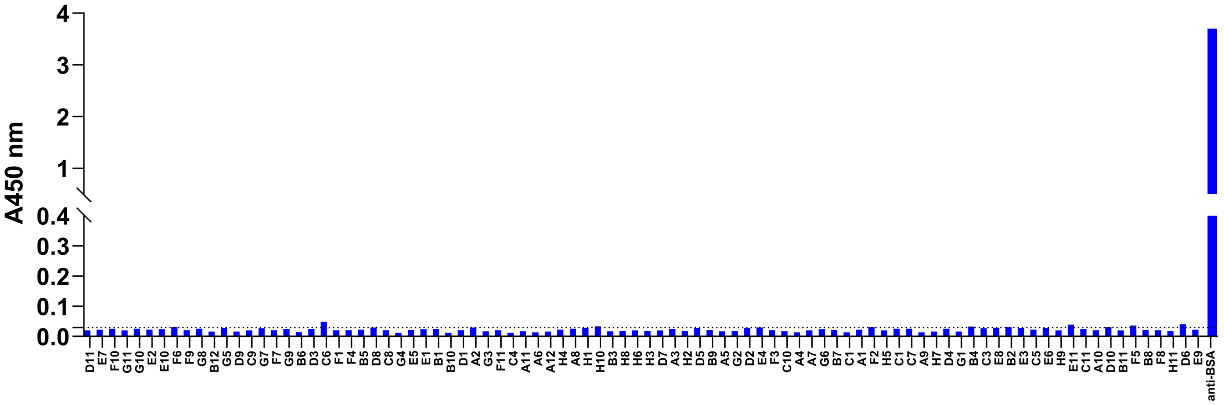

Suppl. Figure 1 Phage ELISA for 90 clones from the third round of panning analyzing the binding to BSA

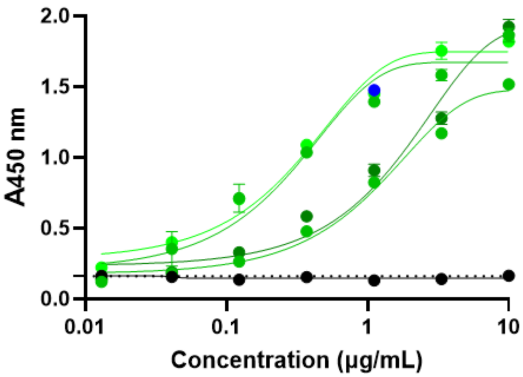

Suppl. Figure 2 Batch-to-batch comparison of the binding of purified scFv D11 to hrCD36 by ELISA

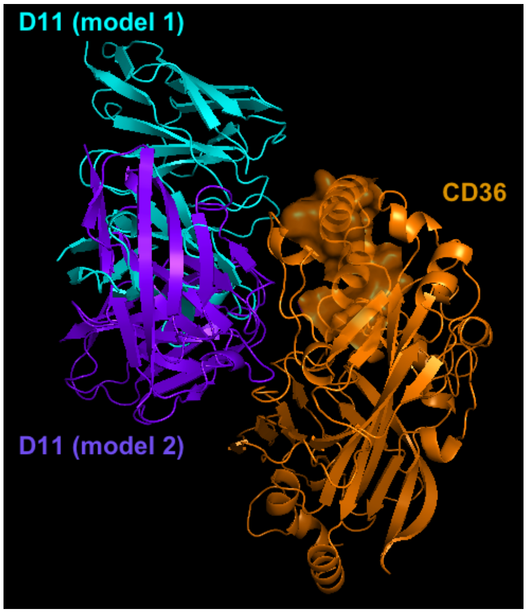

Suppl. Figure 3: Predicted binding modes for D11 on CD36. The D11 antibody was modeled using the SAbPred server (73) and then docked onto CD36 (PDB ID: 5LGD) using the ClusPro software (74) in antibody mode. Two distinct binding models for D11 (cyan and purple) on CD36 (orange) were identified. Analysis of the interacting residues using the Prodigy server (75) revealed that both D11 models interact with a common region of CD36 comprising residues Arg183-Thy202. The oxLDL binding site on CD36 is depicted as a translucent surface

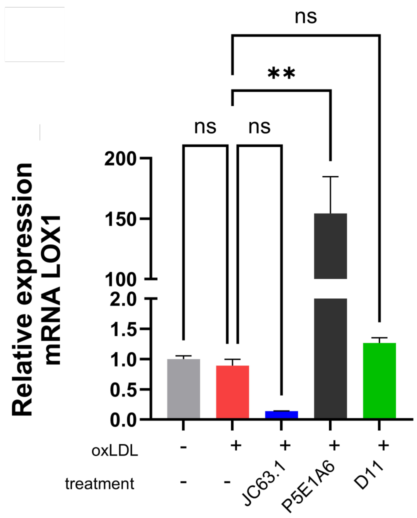

Suppl. Figure 4 RT-qPCR for the quantification of LOX-1 mRNA expression in THP-1 differentiated to foam cells

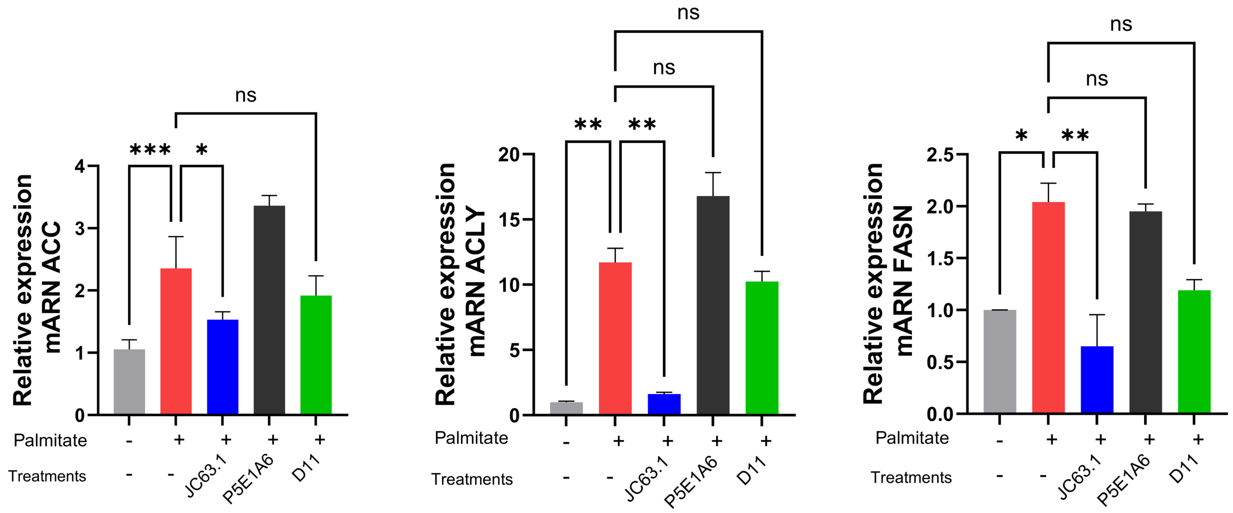

Suppl. Figure 5 RT-qPCR for the quantification of the indicated mRNAs in HepG2 cells exposed to palmitate

Supplementary Table 1 Primers employed for qPCR.

Primer	*Sequence*	*Reference*
CD36-Forward	GGACATACTTGGATATTGAACC	[52]
CD36-Reverse	ACACCAACACTGAGTAAGAT	
SR-A1-Forward	CCTTTACCTCCTCGTGTTT	
SR-A1-Reverse	TGTTGCTCATGTGTTCCA	
LOX-1-Forward	TCTGACCTCCTAACACAAGA	
LOX-1-Reverse	AGATTCTGGTGGTGAAGTTC	
ACAT1-Forward	CGCTGCTGTAGAACCTATT	
ACAT1-Reverse	CCGTATTCTCCTTGCTTCA	

Supplementary references

73. Dunbar J, Krawczyk K, Leem J, Marks C, Nowak J, Regep C, Georges G, Kelm S, Popovic B, Deane CM. SAbPred: a structure-based antibody prediction server. *Nucleic Acids Res* (2016) 44:W474–W478. doi: 10.1093/NAR/GKW361

74 Brenke R, Hall DR, Chuang G-Y, Comeau SR, Bohnuud T, Beglov D, Schueler-Furman O, Vajda S, Kozakov D. Application of asymmetric statistical potentials to antibody–protein docking. *Bioinformatics* (2012) 28:2608–2614. doi: 10.1093/bioinformatics/bts493

75 Xue LC, Rodrigues JP, Kastritis PL, Bonvin AM, Vangone A. PRODIGY: a web server for predicting the binding affinity of protein–protein complexes. *Bioinformatics* (2016) 32:3676–3678. doi: 10.1093/bioinformatics/btw514
